# Simultaneous live-imaging of peroxisomes and the ER in plant cells suggests contiguity but no luminal continuity between the two organelles

**DOI:** 10.3389/fphys.2013.00196

**Published:** 2013-07-24

**Authors:** Kiah Barton, Neeta Mathur, Jaideep Mathur

**Affiliations:** Laboratory of Plant Development and Interactions, Department of Molecular and Cellular Biology, University of GuelphGuelph, ON, Canada

**Keywords:** peroxisome, ER, drp3a, membrane-contact-sites, live-imaging, EosFP

## Abstract

Transmission electron micrographs of peroxisomes in diverse organisms, including plants, suggest their close association and even luminal connectivity with the endoplasmic reticulum (ER). After several decades of debate *de novo* peroxisome biogenesis from the ER is strongly favored in yeasts and mammals. Unfortunately many of the proteins whose transit through the ER constitutes a major evidence for peroxisome biogenesis from the ER do not exhibit a similar localization in plants. Consequently, at best the ER acts as a membrane source for peroxisome in plants. However, in addition to their *de novo* biogenesis from the ER an increase in peroxisome numbers also occurs through fission of existing peroxisomes. In recent years live-imaging has been used to visualize peroxisomes and the ER but the precise spatio-temporal relationship between the two organelles has not been well-explored. Here we present our assessment of the peroxisome-ER relationship through imaging of living *Arabidopsis thaliana* plants simultaneously expressing different color combinations of fluorescent proteins targeted to both organelles. Our observations on double transgenic wild type and a drp3a/apm1 mutant Arabidopsis plants suggest strong correlations between the dynamic behavior of peroxisomes and the neighboring ER. Although peroxisomes and ER are closely aligned there appears to be no luminal continuity between the two. Similarly, differentially colored elongated peroxisomes of a drp3a mutant expressing a photoconvertible peroxisomal matrix protein are unable to fuse and share luminal protein despite considerable intermingling. Substantiation of our observations is suggested through 3D iso-surface rendering of image stacks, which shows closed ended peroxisomes enmeshed among ER tubules possibly through membrane contact sites (MCS). Our observations support the idea that increase in peroxisome numbers in a plant cell occurs mainly through the fission of existing peroxisomes in an ER aided manner.

## Introduction

Single membrane bound, spherical-ovoid 0.1–1.5μm organelles that appeared closely associated with the ER were first reported at the ultra-structural level in the proximal convoluted tubules of mouse kidney by Rhodin (1954) and in hepatic parenchymal cells by Rouiller and Bernhard (1956). Soon after these microbodies were reported in plants (Porter and Caulfield, 1958; Mollenhauer et al., 1966; Frederick et al., 1968; Frederick and Newcomb, 1969). The discovery that certain microbodies contain catalase and other hydrogen-peroxide producing oxidases resulted in their being named peroxisomes (de Duve and Baudhuin, 1966; de Duve, 1969). Peroxisomes are now recognized as major producers as well as scavengers of reactive oxygen and nitrogen species (del Río et al., 2003; Palma et al., 2009).

While the biochemical characterization of peroxisomes progressed rapidly (reviewed in Tolbert and Essner, 1981) their cell biological dissection also kept pace through the development of several cytochemical procedures for staining peroxisomes using 3,3′ diaminobenzidine (DAB; Graham and Karnovsky, 1966; Fahimi, 1969; Hirai, 1969; Novikoff and Goldfischer, 1969). The DAB staining method localized the catalase in peroxisomes and greatly facilitated their identification in cells, and the evaluation of their relative abundance and relationship to other organelles, specially the endoplasmic reticulum (ER) (Frederick and Newcomb, 1969; Vigil, 1970).

The microbody–ER association noted in early electron microscopy based studies [reviewed by Hruban and Rechcigl (1969)] suggested that the semi-opaque material characteristic of microbodies is deposited within dilated portions of the ER that enlarge to form microbodies (Novikoff and Shin, 1964). Indeed ER like projections and continuities were observed so often in the rat liver cells that Novikoff and Shin (1964) considered microbodies to be always attached to the ER. Observations on absorptive cells of the mammalian small intestine also allowed small peroxisomes called micro-peroxisomes to be considered as localized dilations of the smooth ER that retain numerous continuities (Novikoff and Novikoff, 1972; Novikoff et al., 1973a). Since peroxisome isolation was a major contributor in their biochemical characterization Novikoff and Novikoff (1972) speculated that the connections between peroxisomes and the ER might be broken during homogenization and subsequent isolation through centrifugation. The combined observations from microscopy and pulse chase experiments resulted in the vesiculation model wherein peroxisome biogenesis was proposed as taking place through a budding mechanism from the ER (Beevers, 1979). Whereas a complete list of publications providing documentation of associations between microbodies/peroxisomes and the ER is not presented here some outstanding contextual publications are Svoboda and Azarnoff (1966), Essner (1967), Essner (1969), Svoboda et al. (1967), Magalhaes and Magalhaes (1971), de Duve (1973), Novikoff et al. (1973a), Novikoff et al. (1973b), Reddy and Svoboda (1973), Frederick et al. (1975) and Hirai et al. (1983). Early studies clearly pointed to an ER based origin and intimate connectivity between peroxisomes and the ER.

However, the realization that peroxisomes do not possess their own DNA or protein synthesis machinery had already made it apparent that most of their membrane and matrix proteins are imported post-translationally from the cytosol (Lazarow and de Duve, 1973; Goldman and Blobel, 1978; Lazarow et al., 1980, 1982; Koster et al., 1986). The possibility that peroxisomes did not have to be created from the ER but could actually be formed from pre-existing peroxisomes was raised (Legg and Wood, 1970). The dominant peroxisome vesiculation model was rigorously tested by Poole et al. (1970), who searched for gradual dilation of ER tubules to form peroxisomes but were unable to find them. Subsequently Lazarow and Fujiki (1985) assessed the existing ultra-structural and morphological evidence as compared to the biochemical information and laid down a stringent criterion that sought direct luminal connectivity between the ER and the peroxisome.

Today there is increasing appreciation that peroxisomes are endomembrane derivatives (South and Gould, 1999; Geuze et al., 2003; Kunau, 2005; Tabak et al., 2008, 2013). It is believed that while *de novo* biogenesis of peroxisomes can occur directly from the ER, existing peroxisomes in a cell can also undergo fission to form more peroxisomes (Motley and Hettema, 2007). These recent molecular genetic and biochemical evidence have been taken into account in recent reviews (Tabak et al., 2003, 2013; Titorenko and Mullen, 2006; Fagarasanu et al., 2007) and resulted in models such as the “ER semi-autonomous peroxisome maturation and replication” for peroxisome biogenesis in plants (Mullen and Trelease, 2006; Trelease and Lingard, 2006) and for yeasts (Titorenko and Rachubinski, 2009). Additional detailed discussion on peroxisome biogenesis can be found in recent reviews by Hu et al. (2012), Tabak et al. (2013), and Theodoulou et al. (2013). The transit and accumulation of specific peroxisomal proteins such as peroxin 16 (pex16) (Kim et al., 2006), pex3 and pex19 (Hoepfner et al., 2005; Kragt et al., 2005), provide convincing evidence that favors peroxisome biogenesis from the ER in yeasts and mammals (van der Zand et al., 2010, 2012; Lam et al., 2010; Agrawal et al., 2011; Theodoulou et al., 2013). However, there is no clear evidence for the formation of peroxisomes directly from the ER in plants (Trelease and Lingard, 2006). The formation of an ER-peroxisome intermediate compartment (ERPIC) has been proposed but its actual relationship with the ER has not been adequately demonstrated (Mullen and Trelease, 2006; Trelease and Lingard, 2006). Despite the early micrographs suggesting ER-microbody associations (Reddy and Svoboda, 1973; Shio and Lazarow, 1981; Gorgas, 1984, 1985; Yamamoto and Fahimi, 1987) at best the ER in plants is viewed as a source of membrane components, which are delivered in some sort of membrane carrier to pre-existing peroxisomes (Titorenko et al., 2000; Mullen and Trelease, 2006; Hu et al., 2012).

While ultrastructural, biochemical and molecular-genetic approaches to understanding the peroxisome-ER link have been commendable, the direct and simultaneous visualization of the two organelles has not been carried out in plants. Nevertheless over the past years many fluorescent protein probes, mainly based on green fluorescent protein (GFP) and its color variants, that highlight peroxisomes and the ER separately have been developed for living plant cells (Mathur, 2007; Illuminated Plant Cell http://www.illuminatedcell.com/cytomembranes.html). Fluorescent highlighting of the 0.4–1.5 μm diameter peroxisomes shows their hitherto unexplained erratic motility that includes stop and go motion, sudden twists and turns including U-turns, and an almost individualistic manner of movement where one peroxisome might remain almost static while others around it move at varying velocities (Collings et al., 2002; Jedd and Chua, 2002; Mano et al., 2002; Mathur et al., 2002; Rodríguez-Serrano et al., 2009). In contrast to microtubule dependent movement of peroxisomes in mammalian cells (Wiemer et al., 1997; Schrader et al., 2003) their motility in plant cells takes place along F-actin strands in a myosin dependent manner (Collings et al., 2002; Jedd and Chua, 2002). By combining fluorescent probes for peroxisomes and the ER into one plant it is possible to look at both organelles in living plant cells simultaneously without the encumbrance and possibility of creating fixation induced artifacts, the need for sectioning and the limitation of single snapshots.

Here we report observations on peroxisomes and the ER obtained through simultaneous visualization of both organelles in double-transgenic plants of *Arabidopsis thaliana*. Our investigations have been extended to two alleles of the *drp3A/apm1* mutant of Arabidopsis (Mano et al., 2004), which offer aberrantly elongated peroxisomes and thus raise the chances of assessing peroxisome-ER connectivity or continuity. The live imaging study is complemented by 3D iso-surface rendering of confocal derived image stacks that provide more insight through a volume rendered version of the digital images. Our observations in living plant cells strongly support peroxisome–ER contiguity but have been unable to find evidence of luminal continuity between two inter-twined peroxisomes as well as between peroxisomes and the ER.

## Results

### The erratic movement of peroxisomes closely simulates the dynamic behavior of neighboring ER tubules and cisternae

As noted earlier by Jedd and Chua (2002), Mano et al. (2002), Mathur et al. (2002) and Collings et al. (2002) the basis for erratic movement of peroxisomes in plants has remained unexplained. The seemingly individualistic patterns of peroxisomal motility include staying in one subcellular location for varying durations, bi-directional movements, cyclic revolution within a small region of the cell, U-turns, and sudden lateral or tangential forays (Mano et al., 2002; Mathur et al., 2002). Much of the straight axial movement can be attributed to direct peroxisomal dependence on myosin motors and the underlying F-actin cytoskeleton as part of the main cytoplasmic stream (Grolig and Pierson, 2000; Jedd and Chua, 2002). However, neither myosin motors nor the existence of fine F-actin tracks directly elucidate the mechanism underlying sudden stops, oscillations and other peculiarities of peroxisomal motility (Mathur et al., 2002). Interestingly the fine cortical F-actin mesh in a plant cell also provides a structural basis for cortical ER organization in plants (Quader and Zachariadis, 2006; Runions et al., 2006; Sparkes et al., 2009) and thus we speculated that observing the simultaneous behavior of peroxisomes and the ER might provide insights on the erratic motility of peroxisomes.

**Figure 1:**
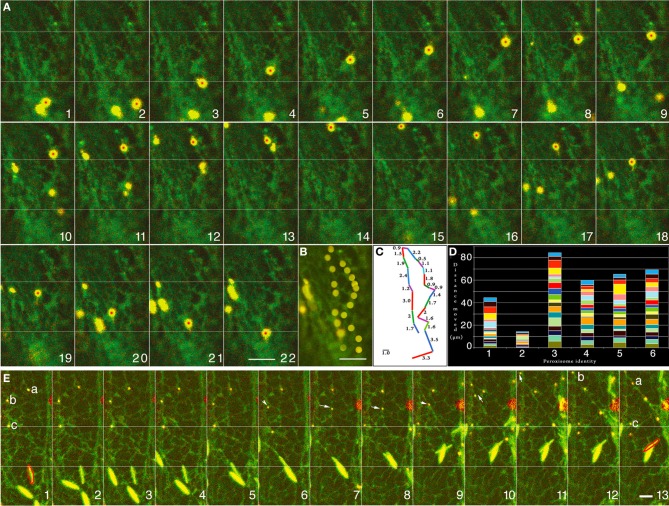
**Representative images of cells from double transgenic Arabidopsis plants expressing peroxisome and ER-targeted probes that suggest correlative behavior of both organelles. (A)** Twenty-two time-lapse images that follows a single YFP-PTS1 highlighted peroxisome (with red dot) against the backdrop of green fluorescent ER (Movie S1). **(B)** A merge of all the frames from “**A**” traces the path of the single peroxisome. Every frame capturing the peroxisome movement has an accompanying subtle change in the subtending ER. **(C)** The movement per frame in the time-lapse image sequence in “**A**” and the path seen in “**B**” displayed as distance moved in μm shows the erratic nature of peroxisome motility. **(D)** Movement of six different peroxisomes followed over 120 s shows the range of variation in their rate of movement and suggests that no two peroxisomes move at the same rate or for the same distance. **(E)** The movement of three peroxisomes (a, b, and c) tracked along with changes in the organization of neighboring ER tubules over 13 sequential images from Movie S2. Frames are separated by ca. 8 s. In contrast the peroxisomes “a” and “b” exhibit oscillations ranging between 2 and 8μm (frames 1–8) alongside short ER-tubules that extend and retract from an ER island. Arrows in frames 6–9 point to the movement of peroxisome “b” wherein the ER tubule harboring it fuses with another tubule to create the familiar ER polygon, whereupon the peroxisome moves again in the pattern defined by the newly organized polygon (frames 7–10). Finally the oscillating peroxisomes “a” and “b” are drawn into a cytoplasmic ER strand and move away rapidly from their previous locations (frames 9–13). Peroxisome “c” shows the least movement (white line across the 13 frames) and remains lodged on a broad patch of ER membrane. An ER body has been outlined in red (bottom half of frames) to provide a comparative estimation of ER reorganization in another area of the cell. Size bars = 2.5μm.

The cortical ER in plant cells displays a dynamic pattern of non-uniform polygons created by anastomosing cyto-membranes wherein smooth tubules and lamellar segments undergo constant rearrangement, sliding, branching and fusion. Sub-cortically, the ER forms long tubules and fenestrated sheets that enmesh different organelles and form a major component of cytoplasmic strands stretching across the large, vacuolated plant cell (Quader and Zachariadis, 2006). Observations on dual marker lines co-expressing ss-mGFP5-HDEL and YFP-PTS1 as well as the YFP-RFP dual combination revealed many instances of forward movement followed by a U-turn with constant variation in the rate of motility. A representative time-lapse series is provided (Figures 1A,B) where the movement of a single peroxisome (Figure 1C—peroxisome with red spot) is charted (Figure 1A frames 1–22) and presented as a merged image (Figure 1B). The variable distance moved between each frame is presented in Figure 1C whereas Figure 1D follows the movement carried out over 120 s for 6 independent peroxisomes. Clearly, different peroxisomes move differently and there is a range of variation in their rate of their movement (Figure 1D). As observed in the images (and the Movie S1) the motility of the peroxisome closely followed the patterns of the neighboring ER (Figure 1A). Another representative series of 13 sequential images (Movie S2) each separated by ca. 8 s (Figure 1E frames 1–13) follows the movements of 3 peroxisomes (Figure 1E; 1-a, b, c) and reveals that to-and-fro oscillations ranging between 2 and 8μm usually occur along short ER-tubules extending and retracting from an ER junction (**1E**; peroxisomes a, b frames 1–8). As shown in Figure 1E (frames 8–10) such movements continue until the ER tubule harboring a peroxisome fuses with another ER tubule to create the familiar ER polygon, whereupon the peroxisome moves again in the pattern defined by the newly organized polygon (Figure 1E; peroxisome b; frames 7–10). Finally the oscillating peroxisomes get drawn into a fast moving cytoplasmic ER strand and move away rapidly from their previous locations at velocities approaching 4 ± 1.5μms^−1^ (Figure 1E frames 9–13).

The cortical ER also consists of large membrane patches that constitute ER islands. When observed using only YFP-PTS1 many peroxisomes appear moribund or execute only Brownian movement (Mathur et al., 2002). When co-visualized with the ER these peroxisomes were confined to the ER islands (e.g., peroxisome-c in Figure 1E). These observations of individual peroxisomal activity within different regions of the interlinked and constantly rearranging ER suggested an explanation for the seemingly erratic peroxisome motility. The observations also suggested how, depending upon the rate of ER motility, up to 65 ± 3% (*n* = 250) peroxisomes within a cell might appear to be arrested in a particular sub-cellular location while other peroxisomes move past them at varying speeds. We further investigated this line of thought by observing peroxisomal motility under conditions that are known to affect ER dynamics.

### Effect of low temperature on ER-peroxisome dynamics

**Figure 2:**
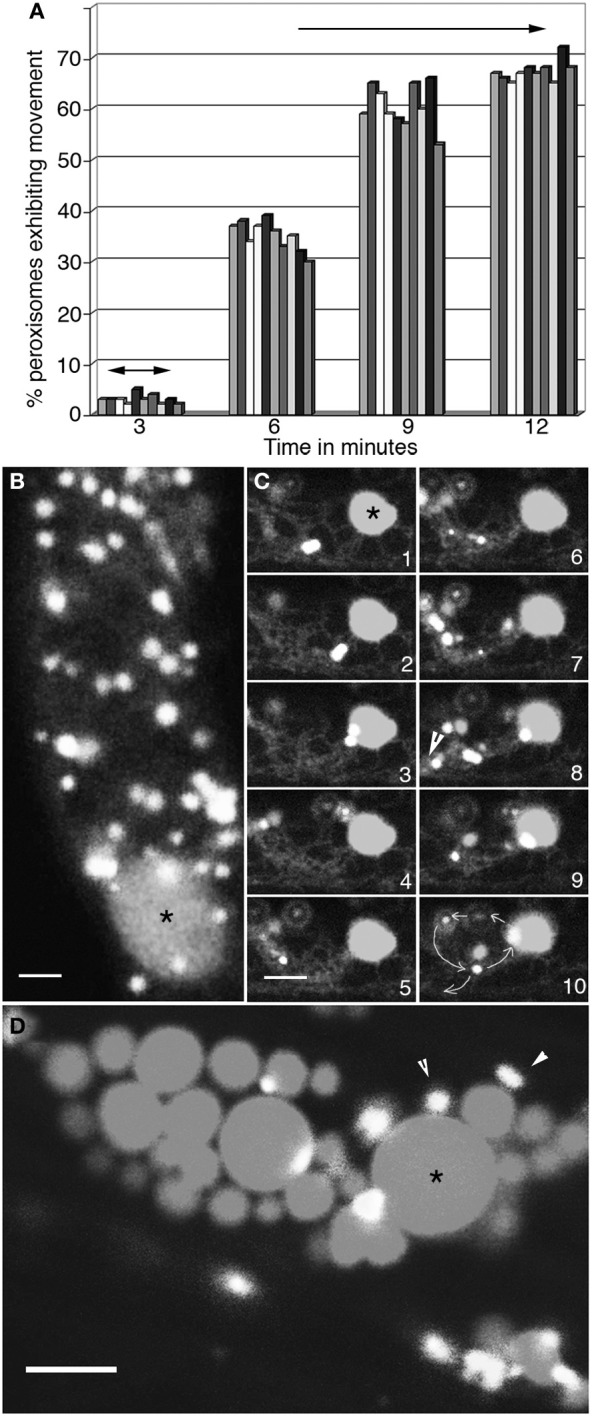
**Concomitant changes take place in peroxisome and ER motility. (A)** Graphical representation of changes in motility of 10 peroxisomes over 12 min as the ambient temperature around cells in 4°C treated seedlings rises to about 23°C. At 3 min following exposure to room temperature peroxisomes begin to exhibit short oscillations (double sided arrows) in synchrony with extension-retraction of ER tubules. The range of movement increases over time (6- and 9-min time points) until by 12 min both the ER and peroxisomes exhibit normal motility including long saltations. **(B)** Both peroxisomes and the ER in cells treated with 1μM latrunculin-B stop moving and large blobs of ER (^*^) surrounded by peroxisomes start appearing. **(C)** Sequential images taken at intervals of after washing away latrunculin-B show the gradual recovery of the ER accompanied by the circumambulation of peroxisomes apparently embedded in the ER (arrowhead in panel 8; path shown by circular arrows in panel 10) during the first 3–4 min. **(D)** Treatment with lat-B for more than 10 min usually leads to the formation of large ER globules (^*^) surrounded by static peroxisomes. Such disorganized ER does not reorganize easily into normal cytoplasmic streaming of organelles. Size bars = 5μm. ^*^indicates an ER globule; arrowheads point to peroxisomes.

Low temperature, around 4°C slows down ER motility, leads to the disappearance of long tubular ER strands and causes a distinct increase in short ER tubules and wider cisternae (Quader et al., 1989). Seedlings expressing RFP-ER and YFP-PTS1 were cold treated for 6 h and analyzed for motility. Figure 2A shows the change in peroxisome motility over 12 min as the ambient temperature around the cold treated seedlings returned to 23°C. Hypocotyl epidermal cells visualized within 3 min of removal from ice-cold temperatures displayed short cortical ER tubules, slow tumbling movements of spindle shaped ER bodies and a low motility of the ER in cytoplasmic strands. Only 2.79 ± 0.9% peroxisomes (*n* = total 100 peroxisomes observed in 10 different cells in 10 seedlings) displayed short oscillations in these cells with the rest exhibiting Brownian movement. By 6 min the number of motile peroxisomes had risen to 35 ± 2.8%, while ER-tubules started exhibiting remodeling and new areas of anastomosis to form the typical polygonal ER mesh. By 12 min the ER exhibited normal cortical and sub-cortical dynamics and 67 ± 5% peroxisomes had achieved motility with normal speeds of 4 ± 1.5μms^−1^ in sub-cortical strands. It was concluded that while slowing down the dynamic behavior of the ER results in slowing peroxisome motility an increase in the movement rate of the ER results in a concomitant change in peroxisomal movement.

### Effect of the actin polymerization inhibitor latrunculin-B

Both actin and myosin inhibitors have previously been shown to interfere with peroxisome (Collings et al., 2002; Jedd and Chua, 2002; Mano et al., 2002; Mathur et al., 2002) and ER (Liebe and Quader, 1994) motility. Both organelles respond similarly by displaying an arrest of motility that is reversible upon washing out the inhibitor. The visualization of their concomitant response to 1μM Latrunculin-B is shown in Figure 2B. In all hypocotyl cells ER and peroxisome motility ceased within 5 min of being exposed to the inhibitor. Both organelles froze in place but did not lose their form. However, in some areas peroxisomes were seen aggregating around large ER blobs (Figure 2B; asterisk). After 10 min the inhibitor was washed away through 5 sequential washes with 10 ml water. Within 5 min of the washing clear indications of ER recovery including the extension-retraction of cortical ER tubules, the resumption of sub-cortical-ER flow in cytoplasmic strands and the stirring of ER bodies, were observed. Concomitantly, as shown through a representative series of time-lapse images the range of peroxisomal oscillations alongside ER tubules started increasing. In this early recovery period covering 3 min 80 ± 6% (*n* = 200) peroxisomes displayed relatively localized movements. These involved multiple circumambulations (path shown in Figure 2C frame 10) with diameters ranging from 10 to 15μm before single peroxisomes moved away on tracks defined by tangential ER tubules (Figure 2C frames 1–10). By 10 min after inhibitor removal a majority of cells displayed normal ER and peroxisomal motility. The drug treatments in our experiments were restricted to 10 min since longer exposures to 1μM Lat-B result in increased ER cisternae and aggregation (Figure 2D) where upon the associated population of peroxisomes becomes limited to Brownian movement. As estimated through observations carried out every 30 min more than 90% of such cells with ER globules did not recover complete organelle motility during 2 h. Similar ER-peroxisome aggregates were often observed in root cells that had been injured or been kept in water, presumably under hypoxic conditions for 2–3 h.

Our observations suggested a close correlation in the pattern of peroxisome motility and the dynamic behavior of ER tubules, but did not provide a sense of the physical relationship between the two organelles. In an earlier study (Sinclair et al., 2009) we have showed that thin tubules, called peroxules, are formed by peroxisomes exposed to subcellular oxidative stress. The peroxules are extended and retracted along ER tubules. We speculated that elongated peroxisomes would provide a better way of understanding the spatio-temporal relationship between peroxisomes and the ER in comparison to our observations on the small ca. 1μm diameter organelles. Peroxisomes in the *apm1/drp3a* (Mano et al., 2004) mutant were visualized for this purpose.

### Unlike ER tubules abnormally elongated peroxisomes in the *apm1/drp3a* mutant do not fuse with each other

**Figure 3:**
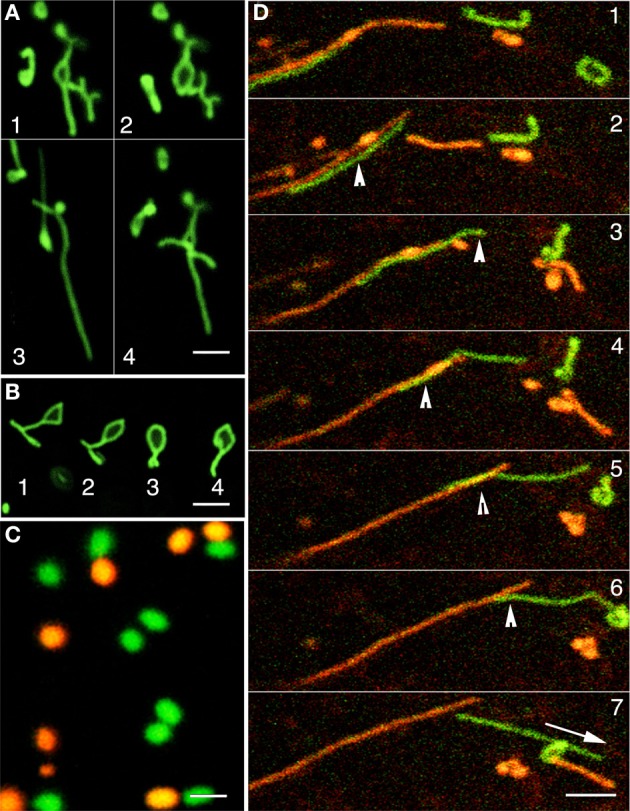
**The dynamic behavior of tubular green fluorescent protein highlighted peroxisomes in the apm1-1 mutant and differential coloring of peroxisomes using a green to red photoconvertible mEosFP. (A)** GFP-highlighted abnormally elongated peroxisomes in the apm1-1 mutant organize into random shapes including open and closed polygons (sequential frames 1–4) within min. **(B)** A single tubular peroxisomes in the apm1-1 morphs sequentially (1–4) into a closed polygon that is highly reminiscent of shapes presented by polygons making up the cortical ER mesh. **(C)** The color of peroxisomes can be changed rapidly from green to red by using the photoconvertible mEos fluorescent protein and irradiating the organelles with violet-blue light. **(D)** A time-lapse image sequence shows the intermingling and subsequent separation of green (non-photo-converted) and red (photo-converted) tubular peroxisomes in a drp3-3 mutant line. The bottom left (Panel 1) shows laterally aligned green and red tubules. In subsequent panels 2–7 the green tubules glides over the red one until the two separate (panel 7). Arrowheads in panels 2–6 shows areas of overlap suggesting close interaction between the tubules. Note that the possible interactions do not appear to result in any exchange of fluorescent proteins. Other smaller tubules morph continuously, seem to interact transiently (panel 3), before separating but do not exchange fluorescent protein either. Scale bar: **(A,B,D)** = 5μm; **(C)** = 2.5μm.

Peroxisomes in the *apm1/drp3a* mutant of *Arabidopsis* are unable to undergo efficient fission and break into the typically spherical, ca. 0.4–1.5μm diameter organelles. Consequently the *apm1* mutants display peroxisomes with an abnormally elongated morphology (Mano et al., 2004). Time lapse imaging of GFP-highlighted elongated peroxisomes in the apm1-1 mutant showed them morphing rapidly into various contorted forms that included polygons and extensions such as those displayed by the ER (Figures 3A,B). However, in the case of the ER the polygons are part of a continuous system and undergo constant fission and fusion (Quader and Zachariadis, 2006; Sparkes et al., 2009). The resemblance of peroxisomes in the apm1-1 mutant to ER polygons and reticulum made us wonder whether the peroxisomal tubules could actually be a part of the existing ER mesh. In such a situation, we reasoned, the tubules would also be able to fuse and exchange luminal proteins. In an earlier report on plastid stromules we have used photo-convertible protein based differential coloring to demonstrate mitochondrial fusion and plastid non-fusion (Schattat et al., 2012). Mitochondrial fusion resulted in their contents mixing and resulted in an intermediate color between red and green (Schattat et al., 2012). Therefore we introduced a green to red photo-convertible EosFP-PTS1 (Sinclair et al., 2009; Figure 3C) in the T-DNA insertional mutant allele of *apm1-1* (SALK_066958). The initially green fluorescent tubular peroxisomes in these lines could be readily photo-converted to red fluorescent ones through irradiation with a violet-blue light (355–425 nm; Figure 3C). We observed occasional fission of single red and green tubules but in 80 tubules from 10 different seedlings, where sometimes multiple tubules intermingled with each other for several min, we were unable to find even a single instance where a red tubular peroxisome fused with a green one. Representative frames from a time-lapse image sequence are presented (Figure 3D). We concluded that individual peroxisomes, whether spherical or tubular, maintain a closed boundary and do not re-establish luminal continuity with other peroxisomes. Having established the individuality and closed nature of tubular peroxisomes, that displayed ER like behavior, we sought to investigate their spatial relationship with ER tubules.

### Elongated peroxisomes in the *apm1/drp3a* and ER tubules display contiguity

**Figure 4:**
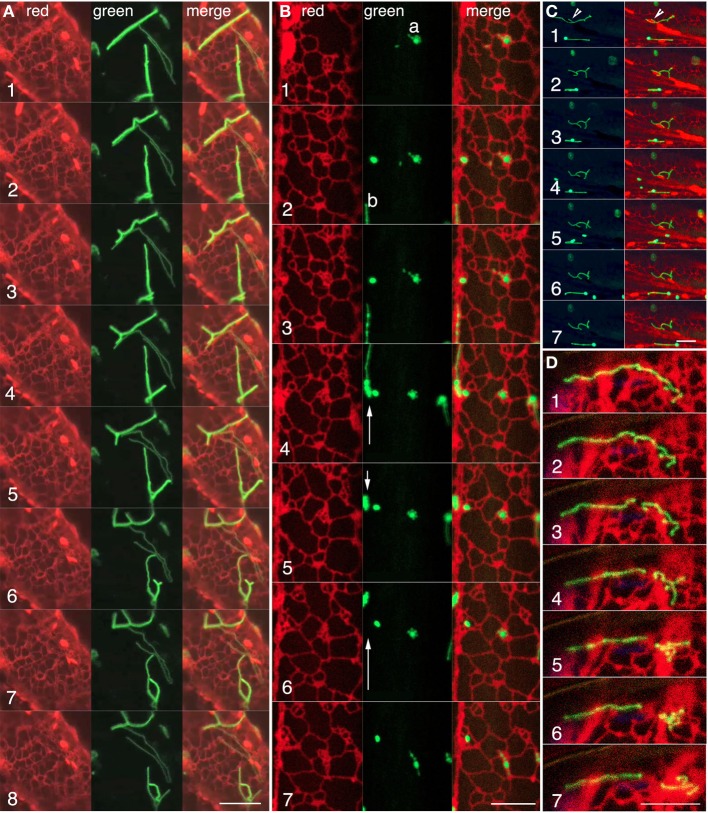
**Confocal visualization of green fluorescent tubular peroxisomes and red fluorescent luminal ER in living hypocotyl cells of the apm1-1 mutant of Arabidopsis. (A)** Representative frames from a time lapse image sequence (Movie S3) showing the correlated behavior of red fluorescent cortical ER tubules around a tubular (green) peroxisome. The tubular peroxisomes lie in an ER lined channel and tubule extension and retraction (frames 2–5), formation of incomplete as well as complete polygonal arrangements (frames 6–8), appear to be defined by the surrounding ER (for animation see Movie S3). **(B)** The behavior of three peroxisome clusters and contiguous ER shows how tubular peroxisomes such as “a” undergo considerable contortions (frames 2, 6–7) while remaining confined to a small region of the ER. During the same period another elongated peroxisome “b” moves forward, retracts and moves again (arrows in green panels) along an ER strand. Note changes in ER organization concomitant with changes in peroxisome behavior (Movie S4). **(C)** A time-lapse sequence showing changes in the morphology of a tubular peroxisome (arrowhead frame 1) due to wrapping (frames 1, 2) and unfolding (frames 3–5) around a spindle shaped ER body and other neighboring ER tubules (see Movie S5). **(D)** Representative sequential images from a time-lapse series showing a tubular peroxisome (frame 1) extending over several ER polygons breaking (frames 2–3) and being pulled apart (frames 4–7) through the reorganization of its neighboring ER. Scale bars = 10μm.

Whereas observations of green fluorescent tubular peroxisomes in the *apm1-1* and its T-DNA insertional allele (SALK_066958) strongly suggested that one is observing an ER like compartment the simultaneous time-lapse observations of peroxisomes and the ER in double transgenics expressing GFP-PTS1 and RFP-ER showed several instances where peroxisomal tubules lay in ER lined channels (Figure 4A; Movie S3). The merged images clearly showed that the extension of tubular peroxisome closely followed the reorganization and dynamics of neighboring ER tubules (Figure 4A; frames 1–8). In other time-lapse images (such as Figure 4B frames 1–7; Movie S4) instances of a tubular peroxisome lodged on an ER island and rejoining a rapidly moving sub-cortical ER strand were noted (Figure 4B; Movie S4). These observations match the observations on motility of spherical peroxisomes in wild type plants (Figures 1A–E) but with the added advantage of being able to observe a larger area in which the two organelles maintain closeness. Occasionally the contiguity may extend to tubular peroxisomes wrapping around ER organelles such as large spindle shaped ER bodies as they pass by as part of the streaming cytoplasm (Figure 4C; Movie S5). In such cases the transient association, even if it occurs against the track being followed originally by the peroxisome (Movie S5), results in the peroxisomal shape tracing out the organelle surface (Figure 4C frames 1–7). In addition several instances were noted where a long tubular peroxisome broke into two unequal parts through the dynamic reorganization of the surrounding ER. The individual bits of tubular peroxisomes drew further apart as the neighboring ER polygons reorganized in different directions (Figure 4D; Movie S6). Observations on the pulling apart and breaking of a tubule through ER reorganization in different directions suggested that the tubular peroxisomes were somehow strongly tethered to the ER and not free to slip out of the ER mesh on their own. A software aided 3D volume rendering was carried out to get more insight into the relationship between the ER and the tubular peroxisomes.

### Tubular peroxisomes appear enmeshed in the endoplasmic reticulum

**Figure 5:**
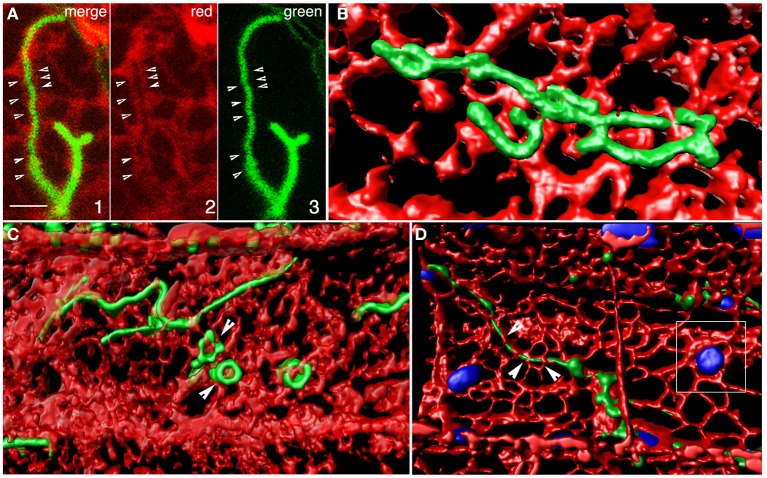
**Iso-surface rendering of confocal image stacks depicting the relationship between tubular peroxisomes and the neighboring ER tubules in the apm1-1 mutant of Arabidopsis. (A)** A region of the cell showing a tubular peroxisome (panels 1, 3) extended within an ER-lined channel (panel 2). Arrowheads point to potential membrane contact sites. Scale bar = 5μm. **(B)** A volume rendered stack of 12 images suggests that the tubular peroxisomes in the apm1-1 mutant are enmeshed and embedded in the ER. Changes in ER organization would be expected to create similar alterations in the morphology of the associated peroxisomal tubule. **(C)** Several tubules threaded between the tubules making u the cortical ER. Note that while some areas of the ER overlap regions of the tubule the tubular peroxisomes can also surround portions of the ER (e.g., arrowhead). **(D)**. A tubular peroxisome interwoven in the cortical ER mesh. Note embedded chloroplasts (blue; outlined square) and the possibility of finding membrane contact sites (arrowheads).

Three-dimensional iso-surface rendering of confocal image stacks showed that peroxisomal tubules are closely inter-twined with tubules of the ER (Figure 5A; Movie S7). Such intertwining probably accounts for their fission as the ER undergoes reorganization in an actin-myosin dependent manner. Their position as embedded tubules in the ER also accounts for their dynamic ER-like behavior. Such coincidental behavior of contiguous ER and tubular peroxisomes suggests that they might have strong adherence to each other. Although the membrane contact sites (MCS) between peroxisomes and the ER are not readily visible in our rendered image the weaving of tubules between ER polygons suggests the possibility of such contact and adhesion sites. However, neither confocal imaging nor the iso-surface rendering suggests luminal connectivity between closed peroxisomal tubules and the ER.

## Discussion

Transmission electron microscopy and cytochemistry based publications in the early phase of research on microbodies/peroxisomes strongly suggested their association with the ER but the inability to actually track peroxisomes being born from the ER drew criticism for the ER-vesiculation model (Legg and Wood, 1970; Poole et al., 1970; Beevers, 1979; Lazarow and Fujiki, 1985). Subsequent thinking considered peroxisomes as independent organelles with a possibly endosymbiont origin (Lazarow and Fujiki, 1985; de Duve, 1996) and radically polarized the field as biologists subscribed to one theory or the other. After more than half a century of debate our views on peroxisomes have come around a full circle and the majority of biologists now subscribe to a kind of status quo model which accepts *de novo* peroxisome biogenesis from the ER as well as the fission of existing peroxisomes as a means of increasing peroxisome numbers in a cell. Given that there are two possibilities for peroxisome proliferation it is likely that certain physiological conditions might favor one over the other. It is equally possible that both phenomena can occur simultaneously. Whereas the present models (Trelease and Lingard, 2006; Titorenko and Mullen, 2006; Fagarasanu et al., 2007; Hettema and Motley, 2009; Mast et al., 2010; Schrader et al., 2012; Tabak et al., 2013) rely heavily on experimental evidence from yeasts and mammals and there is general agreement that the ER plays an important role in the peroxisomal life cycle it is noteworthy that there is no clear proof for peroxisome biogenesis from the ER in plants (Trelease and Lingard, 2006; Hu et al., 2012). Contrary to the convincing evidence of proteins such as the human peroxin16 labeling the entire ER in a cell upon over-expression (Kim et al., 2006), the plant pex16 homolog (e.g., Arabidopsis SSE1/At2g45690; Lin et al., 1999) does not localize in a similar manner (Karnik and Trelease, 2005, 2007). Instead AtPex16-GFP fusion protein accumulates in the form of reticulo-circular tubules that along with similar localization patterns for ascorbate peroxidase (APX; Mullen et al., 1999) are considered to suggest a pre-peroxisomal domain of the ER (Karnik and Trelease, 2005, 2007). Similar ER-like patterns are described by aberrantly elongated peroxisomes in the *apm1/drp3a* mutants of Arabidopsis (Mano et al., 2004). On the contrary, alignment of thin peroxules and the ER were observed and also suggested ER-like shapes (Sinclair et al., 2009). The rapid formation of peroxules and tubular peroxisomes gave rise to the conjecture that a subset of peroxisomes might exist as tightly pinched domains of the ER. It was speculated that the pinching activity might relax under certain stress conditions to allow peroxisomal contents to flow into connected tubules (Mathur, 2009). Here we used the hitherto unexploited technique of simultaneous live imaging of the two organelles to actually observe their relationships.

Our observations on cells with differently colored peroxisomes and the ER clearly show that irrespective of their morphology, peroxisomes closely align with the ER. Indeed when one considers the 3-dimensional aspect of a cell then peroxisomes can be considered enmeshed in the ER. This impression is reinforced through 3D volume rendering of image stacks (Figure 5) and provides a reasonable explanation for the erratic motility of peroxisomes observed in earlier studies (Jedd and Chua, 2002; Mathur et al., 2002). As shown by us, a slowing down of ER motility through cold treatment or treatment with an actin polymerization inhibitor results in a similar slow down of peroxisomes. Conversely an increase in ER motility is also matched by peroxisomes. Whereas observations on spherical peroxisomes and the ER mainly provided an idea of their correlated motility they did not provide a clear idea of spatial relationship. However, the use of tubular peroxisomes in the *apm1/drp3a* mutant clearly shows the intertwining of the two organelles and regions where the ER overlaps or surrounds the peroxisomes (Figures 4, 5). As seen for plastids and mitochondria the ER-mesh around organelles acts as both reinforcement as well as a conduit for trafficking of proteins and lipids. Many such points of interaction between organelles are created through MCS (Levine, 2004; Michel and Kornmann, 2012). Although MCSs similar to those suggested for plastids and mitochondria have not been observed so far between peroxisomes and the ER it is known that these two organelles share a considerable degree of membrane homology (Schlüter et al., 2006) and therefore might have high chances of transient membrane contacts being formed. The presence of MCS with the ER also accounts for the fact that the motility of many organelles in the cytoplasm appears very similar. In this context while considering the strong correlations between the ER and peroxisome activity this study does not overlook the fact that in plants the motility of both peroxisomes and the ER depends upon an acto-myosin system (Jedd and Chua, 2002; Hashimoto et al., 2005; Li and Nebenfuhr, 2007; Peremyslov et al., 2008, 2012; Ueda et al., 2010). Whether the coincidental behavior of the two organelles occurs along separate F-actin strands and involves independent motor molecules is unclear at this stage. There is also the possibility that both organelles become associated with their respective motors but use the same F-actin strands and bundles for movement. An experimental approach that focuses on unraveling the relationship between myosin motors, peroxisomes and the ER is being developed presently and will be reported independently.

The entanglement of peroxisomes in the ER mesh has an additional implication. This involves the moving apart of peroxisomes their fission in a sequential Pex11-DRP3A-DRP3B-FIS1A-FIS1B (Kobayashi et al., 2007; Zhang and Hu, 2009) mediated manner. Peroxisome attachment to the neighboring ER is apparently sufficiently strong to allow pieces of peroxisomes to be pulled apart as the ER polygons reorganize and separate from each other. While we are devising new fluorescent protein based tools to test this phenomenon it is clear that each separated fragment can continue growth by attracting fresh peroxisomal components from the surrounding cytoplasm. Thus our observations suggest that the ER, perhaps in its motor driven state provides the force to allow peroxisomes to undergo fission at a weak point. The weak point where breakage would be favored can be formed through the constrictase activity of a dynamin related protein and other helper proteins (Zhang and Hu, 2009; Hu et al., 2012).

Our present observations on co-visualized peroxisomes and the ER suggest contiguity but do not indicate any signs of luminal continuity between the two organelles. The observations favor the fission of existing peroxisomes in an ER aided manner to increase their numbers in a plant cell. Does this create a problem with the existing models that consider both *de novo* biogenesis as well as fission of existing peroxisomes for increasing the peroxisomal population? Where do the peroxisomes for fission come from in plant cells? The limitation of our present fluorescent protein based tools approach for answering this question is that the probes used for visualizing peroxisomes consist of matrix targeted fluorescent proteins. While existing peroxisomes are reliably highlighted through them these probes would not be expected to highlight precursors of peroxisomes on the ER or elsewhere in the cytoplasm. Moreover, several lines of evidence point that pre-peroxisomal ER derived vesicles (Titorenko et al., 2000) that might be biochemically distinct (van der Zand et al., 2012; Tabak et al., 2013) can fuse together in the general cytoplasm to assemble a peroxisome. If this is indeed the situation then the ER in plants can only be viewed as a source of membrane components and might not be the physical location for *de novo* peroxisome biogenesis.

## Materials and methods

### Generation of fusion constructs and transgenic plants

The creation of constructs YFP-PTS1 (Mathur et al., 2002), mEosFP-PTS1 (Sinclair et al., 2009), ss-RFP-HDEL (Sinclair et al., 2009) has been described earlier. The *apm1-1* mutant line carrying a GFP-PTS1 has been described (Mano et al., 2004) and was used as provided. Seeds of *drp3A-3* (SALK_066958) seeds were obtained from the Arabidopsis Biological Research Center (The Ohio State University, Columbus). Stable transgenic lines of wild type Arabidopsis and the apm1-1 mutant expressing one and two targeted fluorescent proteins were created through the floral dip method (Clough and Bent, 1998), and through crossings.

Arabidopsis plants in sterile culture were grown in petri dishes in an incubator maintained at 21 ± 2°C and a 16/8-h light/dark regime using cool-white light of approximately 80–100μmol m^−2^ s^−2^. To break dormancy *A. thaliana* seeds were incubated at 4°C for 48 h Growth medium for *A. thaliana* WT and mutant seedlings consisted of 1% agar-gelled Murashige and Skoog (1962) basal medium containing Gamborg vitamins (M404; PhytoTechnology labs) supplemented with 3% sucrose (pH 5.8). For obtaining etiolated *A. thaliana* seedlings, petri dishes were wrapped in two layers of aluminum foil right after the cold treatment and a pinhole created in the foil before placing the plates in an upright position. Seedlings were used between 8 and 11 days after sowing on plates for all experiments.

### Microscopy and image rendering

Plant tissue and seedlings were mounted in tap water on a glass depression slide and placed under a coverslip. For plants expressing the photoconvertible mEosFP-PTS1 protein the photoconversion time was varied according to the brightness of the respective organelles. In general, exposure times for peroxisomes were between 3 and 6 s and resulted in bright red organelles. The light source for photo-conversion was a HBO 100 W/2 Mercury Short Arc lamp and the Leica fluorescence filter set “D” (Excitation filter: 355–425 nm; Dichromatic mirror 455 nm; Suppression filter LP 470 nm). The epi-fluorescence setup consisted of a Leica DM-6000CS microscope. Photo-conversion was performed manually by controlling the diaphragm as described earlier (Mathur et al., 2010). Simultaneous imaging of peroxisomal and ER probes was carried out using a Leica TCS-SP5 confocal laser-scanning unit equipped with a 488 nm argon and a 543 nm helium-neon laser. All images were captured using at a color depth of 24bit RGB.

All images and movies were cropped and processed for brightness/contrast as complete image or stacks using either Adobe Photoshop CS3 (http://www.adobe.com) or the ImageJ distribution Fiji (http://pacific.mpi-cbg.de/wiki/index.php/Fiji). Adobe Photoshop was used for annotation of movies. Imaris software (v. 6.4.0; Bitplane AG) was used to render iso-surface 3 D rendering of ER and peroxisomes from confocal image stacks and x-y-time series. All experiments were carried out at least five times.

### Conflict of interest statement

The authors declare that the research was conducted in the absence of any commercial or financial relationships that could be construed as a potential conflict of interest.

## Supplementary material

Movie S1 **Time-lapse sequence of 26 frames taken 1 s apart (played at 2X speed) showing simultaneously a peroxisome and the subtending ER suggests correlations between the movement of the two organelles**. Note how the peroxisome moves forwards and then returns to nearly the same position after making a U-turn. (Basis for Figures 1A–C).

Movie S2 **Time-lapse sequence of 39 frames taken 1 s apart showing three different peroxisomes co-visualized with the ER**. The sequence depicts how ER organization has a direct effect on peroxisomal positioning and relocation. (Basis for Figure 1E).

Movie S3 **Sequential time-lapse comprising 120 images taken at about 3 s intervals played over 50 s show red fluorescent ER tubules harboring green fluorescent tubular peroxisomes in the apm1-1 mutant of Arabidopsis**. The time-lapse sequence shows tubule extension, retraction, branching, and polygon formation in tandem with contiguous ER tubules. At some stages the tubule appears to move within an ER lined channel. (Basis for Figure 4A).

Movie S4 **A 33 s time-lapse sequence comprising 165 frames of tubular peroxisomes and neighboring ER in the apm1-1 mutant of Arabidopsis suggests the ER as the basis for holding a peroxisome cluster in a particular location as well as its unfolding and extension**. (Basis for Figure 4B).

Movie S5 **Co-visualization of RFP-highlighted ER and GFP-highlighted tubular peroxisomes in the apm1-1 mutant suggests how the shape of a peroxisome is molded through its transient adhesion with ER structures such as spindle shaped ER bodies and ER polygons**. Note that the flow of the ER carrying the ER body is against the direction of flow for the tubular peroxisome, suggesting that transient contacts can occur even between organelles that are moving away from each other. (160 frames taken over 10 min played at 5× speed) (Basis for Figure 4C).

Movie S6 **Co-visualization of ER and tubular peroxisomes in the apm1-1 mutant show how long tubules might be broken and then draw apart through the reorganization of neighboring cortical ER**. (40 frames covering ca. 3.5 min) (Basis for Figure 4D).

Movie S7 **An iso-surface rendering of a confocal image stack provides a 3D impression of green tubular peroxisomes enmeshed and embedded in the red colored cortical ER**. (Basis for Figure 5B).
